# Supplementary material for: High titre neutralizing antibodies in response to SARS–CoV–2 infection require RBD–specific CD4 T cells that include proliferative memory cells
Source: Front Immunol. 2022 Dec 5;13:1032911. doi: 10.3389/fimmu.2022.1032911 (PMC9762180; doi:10.3389/fimmu.2022.1032911)
Supplement: Supplementary file 1 [file DataSheet_1.pdf]

Supplementary Table 1- *ADAPT study patient characteristics.* Age, gender, ethnicity, and severity at month 3 and 8 post-infection.

Characteristics	ADAPT Cohort				
	Recombinant RBD 3 month OX40 screen	Antibody Low Convalescent	Antibody Low Vaccinated	Antibody High Convalescent	Antibody High Vaccinated
n	25	12	14	12	20
Mean age (y)	42 ± 10.8	47 ± 14.3	54 + 17.9	54 ±16.1	59 ± 13.8
Male gender, n (%)	12 (48%)	6 (50%)	9 (64%)	12 (100%)	12 (60%)
Ethnicity (n, %-total)					
- African American	0 (0%)	0 (0%)	0 (0%)	0 (0%)	0 (0%)
- Asian	2 (8%)	0 (0%)	1 (7%)	0 (0%)	0 (0%)
- Caucasian	19 (76%)	10 (83%)	13 (93%)	12 (100%)	20 (100%)
- Hispanic	2 (8%)	2 (17%)	0 (0%)	0 (0%)	0 (0%)
- Others	2 (8%)	0 (0%)	0 (0%)	0 (0%)	0 (0%)
Median Days post infection					
3 months (FU3)	76 ± 18	74.5 ± 15	68 ± 19	62.5 ± 14	46.5 ± 25
8 months (FU5)	228 ± 60	228 ± 75	250 ± 30	226.5 ± 24	244 ± 69
Vaccination 2nd dose			528 ± 26		479 ± 87
Severity (n, %-total)					
- Mild	9 (36%)	6 (50%)	6 (42.85%)	2 (16.7%)	9 (45%)
- Moderate	16 (64%)	6 (50%)	6 (42.85%)	7 (58.3%)	8 (40%)
- Severe	0 (0%)	0 (0%)	2 (14.3%)	3 (25%)	3 (15%)

Supplementary Table 2. Peptide pools: SN (spike non-RBD), SR (spike RBD), NP (Nucleocapsid protein).

#	Peptide	Amino acid sequence
1	SN1	NNATNVVIKVCEFQF
2	SN2	CTFEYVSQPFLMDLE
3	SN3	TRFQTLLALHRSYLT
4	SN4	LLALHRSYLTPGDSS
5	SN5	SVASQSIIAYTMSLG
6	SN6	SIIAYTMSLGAENSV
7	SN7	NLLLQYGSFCTQLNR
8	SN8	TQLNRALTGIAVEQD
9	SN9	NFSQILPDPSKPSKR
10	SN10	KPSKRSFIEDLLFNK
11	SN11	SFIEDLLFNKVTLAD
12	SN12	TDEMIAQYTSALLAG
13	SN13	AQALNTLVKQLSSNF
14	SN14	VQIDRLITGRLQSLQ
15	SN15	SLLIVNNATNVVIKV
16	SN16	CEFQFCNDPFLGVYY
17	SN17	IGINITRFQTLLALH
18	SN18	FTVEKGIYQTSNFRV
19	SN19	AYSNNSIAIPTNFTI
20	SN20	VFAQVKQIYKTPPIK
21	SN21	CAQKFNGLTVLPPLL
22	SN22	AQYTSALLAGTITSG
23	SN23	WTFGAGAALQIPFAM
24	SN24	GAISSVLNDILSRLD
25	SN25	APHGVVFLHVTYVPA
26	SN26	ELDKYFKNHTSPDVD
27	SN27	GINASVVNIQKEIDR
28	SN28	LNEVAKNLNESLIDL
29	SN29	YEQYIKWPWYIWLGF
30	SR1	GIYQTSNFRVQPTES
31	SR2	SNFRVQPTESIVRFP
32	SR3	QPTESIVRFPNITNL
33	SR4	IVRFPNITNLCPFGE
34	SR5	CPFGEVFNATRFASV
35	SR6	VFNATRFASVYAWNR
36	SR7	YAWNRKRISNCVADY
37	SR8	GVSPTKLNDLCFTNV
38	SR9	GKIADYNYKLPDDFT
39	SR10	YNYKLPDDFTGCVIA
40	SR11	GCVIAWNSNNLDSKV
41	SR12	GGNYNYLYRLFRKSN
42	SR13	YLYRLFRKSNLKPFE
43	SR14	FNCYFPLQSYGFQPT
44	NP1	MSDNGPQNQRNAPRITF
45	NP2	NQRNAPRITFGGPSDSTG
46	NP3	DDQIGYYRRATRRIR
47	NP4	MKDLSPRWYFYYL
48	NP5	LSPRWYFYYLGTGPEAGL
49	NP6	LLESELVIGAVILRGHLR
50	NP7	DAALALLLLDRLNQL
51	NP8	LLLLDRLNQLESKMS
52	NP9	AFFGMSRIGMEVTPSGTW
53	NP10	GMEVTPSGTWLTYTGAIK
54	NP11	PSGTWLTYTGAIKLD
55	NP12	TWLTYTGAIKLDDKDPNF
56	NP13	PNFKDQVILLNKHIDAYK
57	NP14	LLNKHIDAYKTFPPTEPK

Supplementary Figure 1

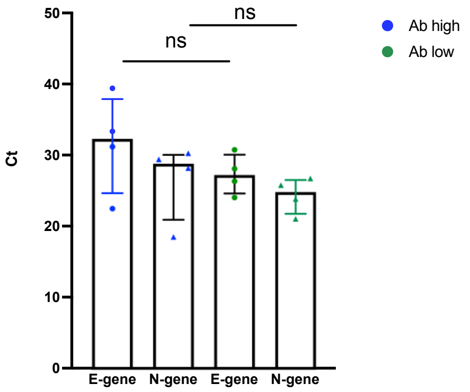

Supplementary Figure 1 – Available Ct values from diagnostic PCR tests
Available Ct values from PCR assays for patients' initial diagnostic testing are shown, using E-gene and N-gene based assays, for Ab high and Ab low subjects, respectively.

Supplementary Figure 2

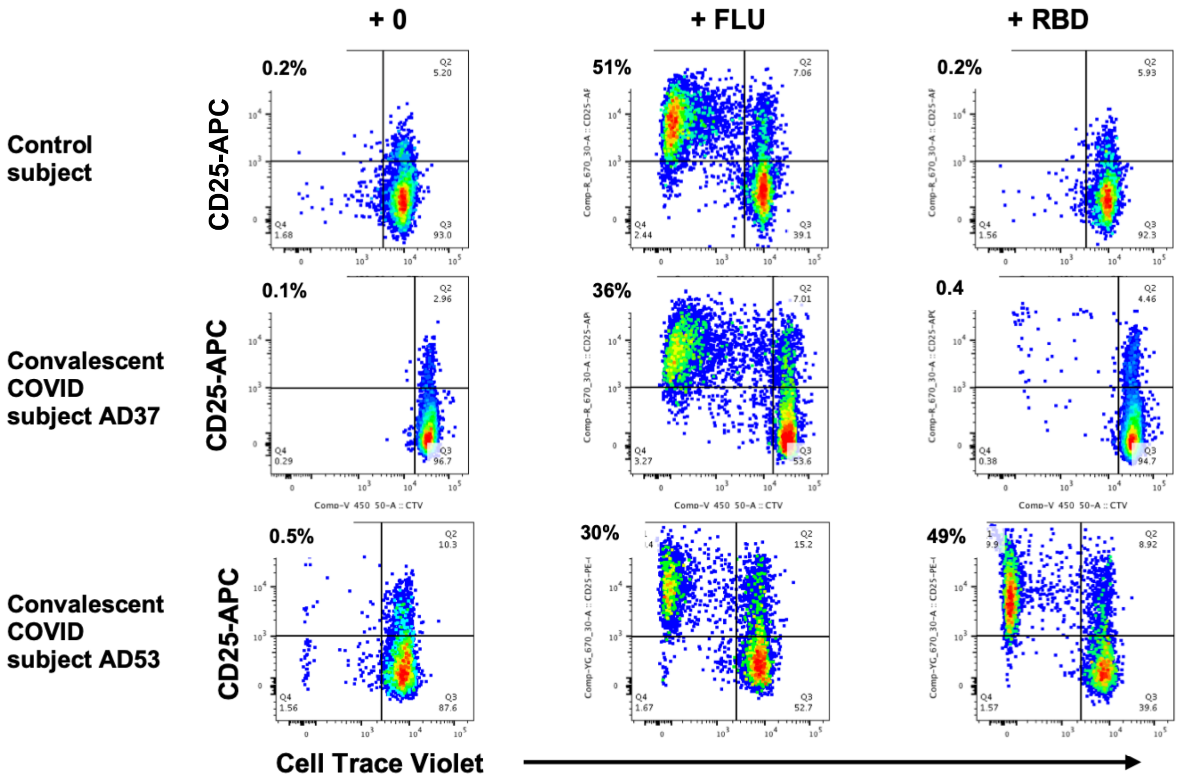

Supplementary Figure 2 – Cell trace violet (CTV) 7-day proliferation assay

Flowplots, gated on CD4 T cells, show representative results of CTV 7-day proliferation assays for PBMC incubated without antigen (negative control; left flowplots), with Flu antigen (positive control; middle flowplots) and with recombinant RBD (right flowplots), for an unexposed control subject (top row), a convalescent ADAPT subject without a detectable RBD proliferation response (middle row) and for a convalescent ADAPT subject with a detectable RBD proliferation response (bottom row)

Supplementary Figure 3

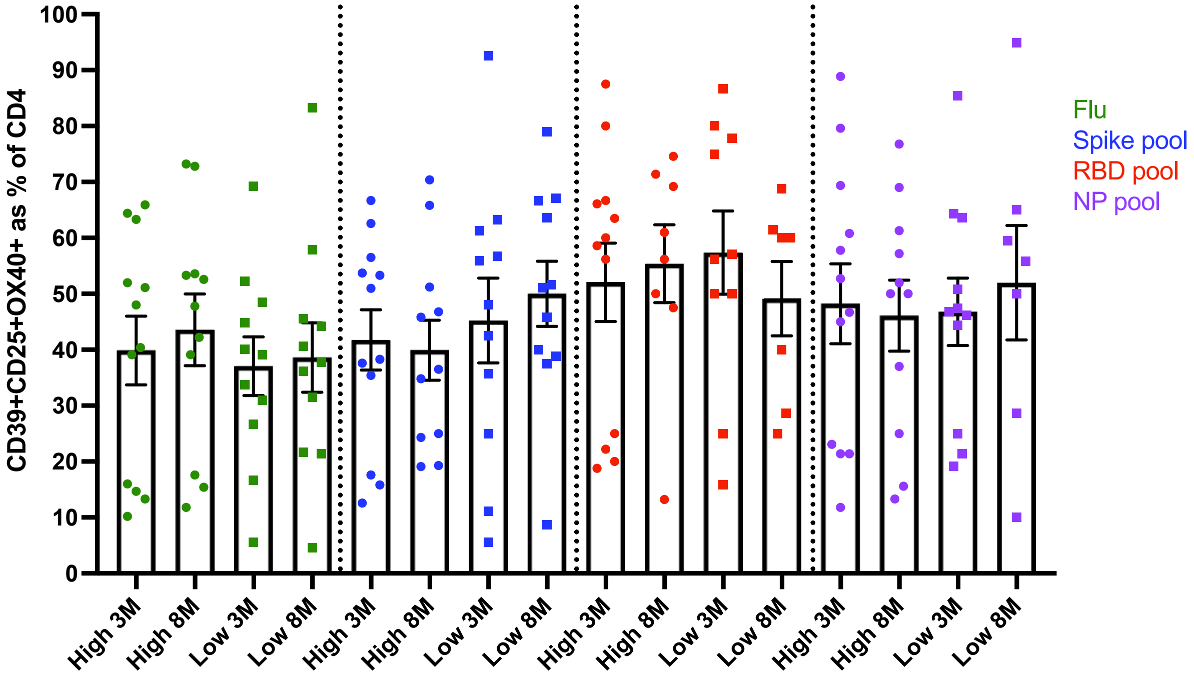

Supplementary Figure 3 - CD39+ antigen-specific Treg responses.

(A) No difference was observed in CD39+ antigen-specific Tregs (CD25+CD134+CD4+) following stimulated with flu or SARS-CoV-2 peptide pools.

Supplementary Figure 4

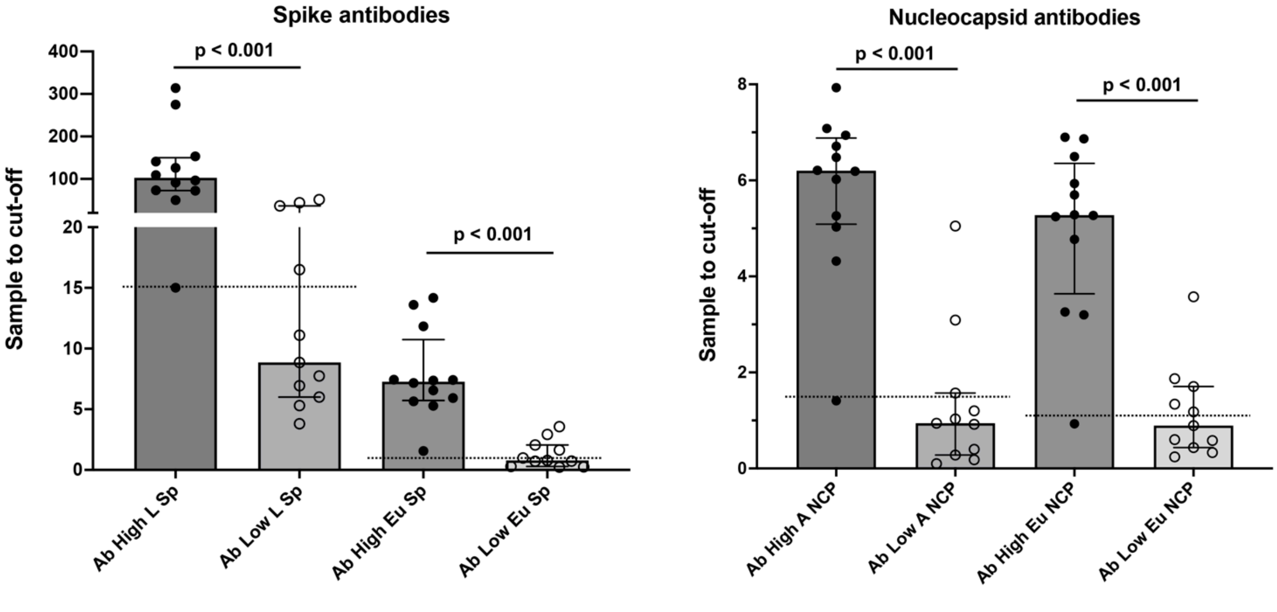

<u>Supplementary Figure 4</u> – Diagnostic assays for Spike and Nucleocapsid antibodies

Serum samples from Ab High subjects and Ab Low subjects were tested for anti-Spike antibodies (Left graph) using Liaison (L) assay and Euroimmune (Eu) assay. The levels of Sample:cut-off ratios for positive results are shown as dashed lines, for each assay.
Also, serum samples from Ab High subjects and Ab Low subjects were tested for anti-Nucleocapsid antibodies (Right graph) using Abbott Architect (A) assay and Euroimmune (Eu) assay. The levels of Sample:cut-off ratios for positive results are shown as dashed lines, for each assay.
Differences between the Ab High and Ab Low subject groups were tested by Mann-Whitney unpaired non-parametric test, and p values for significant differences are shown.

Supplementary Figure 5

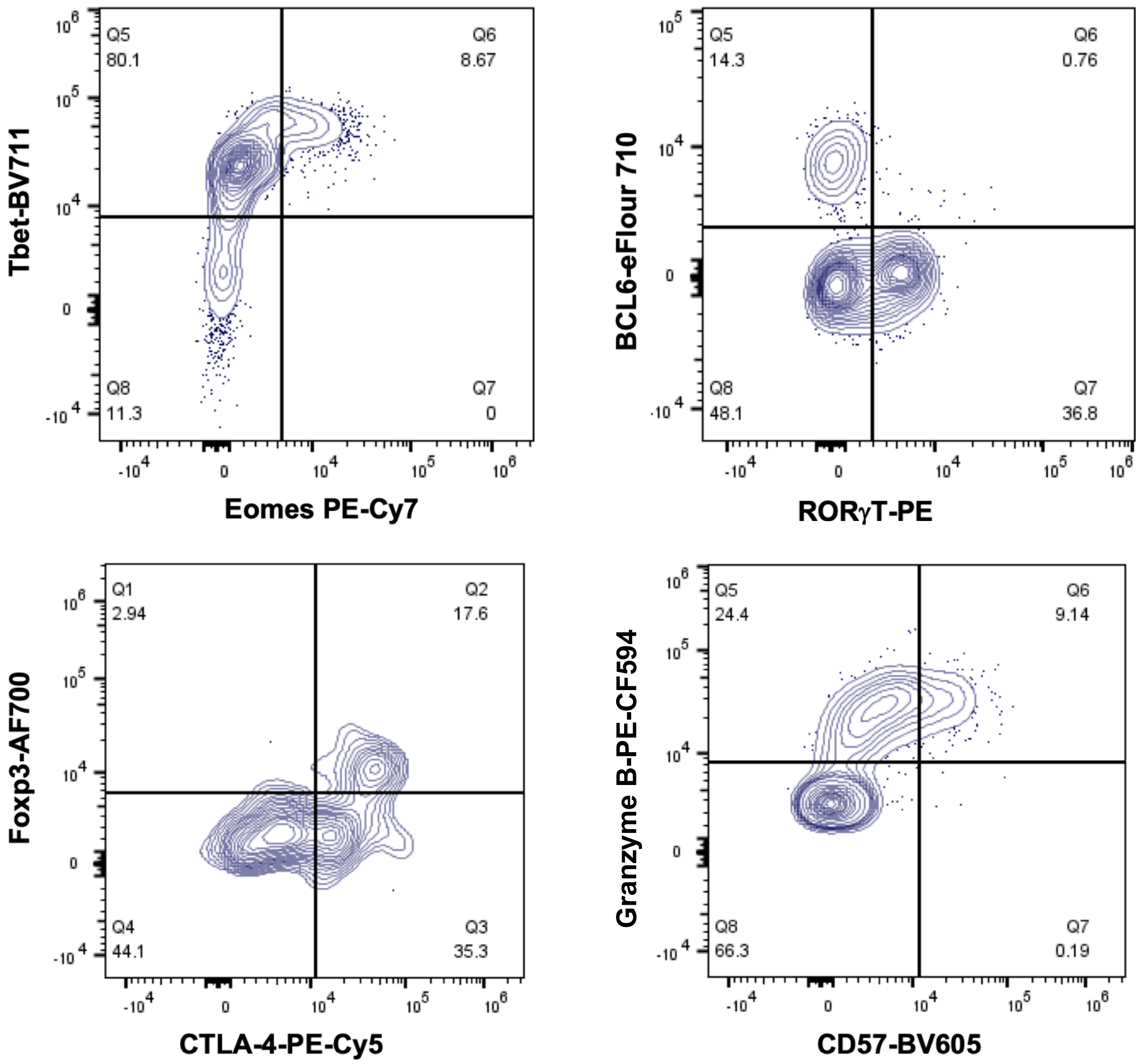

<u>Supplementary Figure 5</u> – Intracellular staining of proliferated CD4 T cells at day 5
Representative flowplots of SEB-stimulated CTVdim proliferated CD4 T cells at day5 showing gating for intracellular phenotyping.

Supplementary Figure 6

(A)

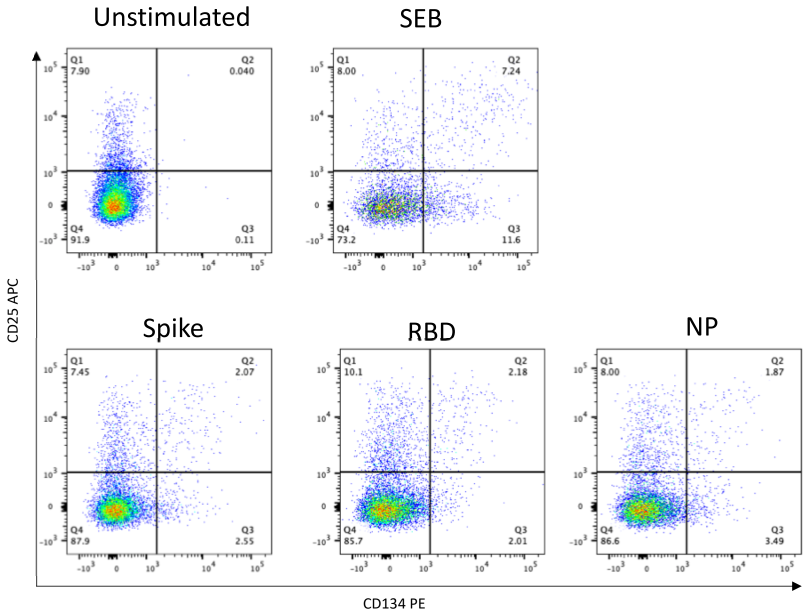

(B)

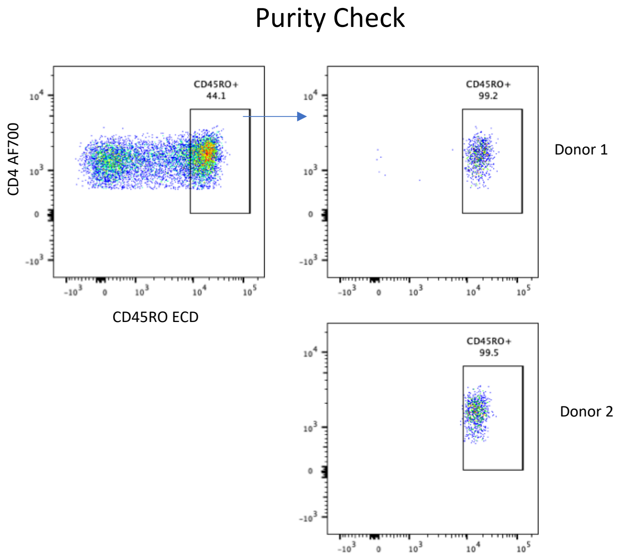

Supplementary Figure 6 - Cell sorting for single-cell transcriptomics.

(A) Representative flowplot of 1 participant shown following 48hr stimulation with SAR-CoV-2 peptide pools, with SEB as a positive control. Antigen-specific CD4+ T cells were bulk sorted from Q2 (CD25+CD134+) on the BD Aria III. (B) CD45RO+ ex vivo (unstimulated) memory CD4+ T cells were also bulk sorted for 10X sequencing. A purity of >99% was achieved for all samples.

Supplementary Figure 7
(A)

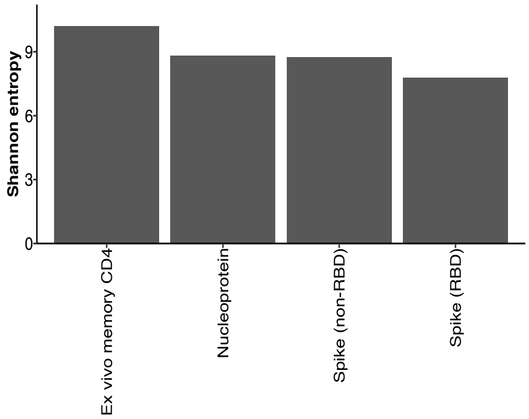

(B) Ex vivo Spike

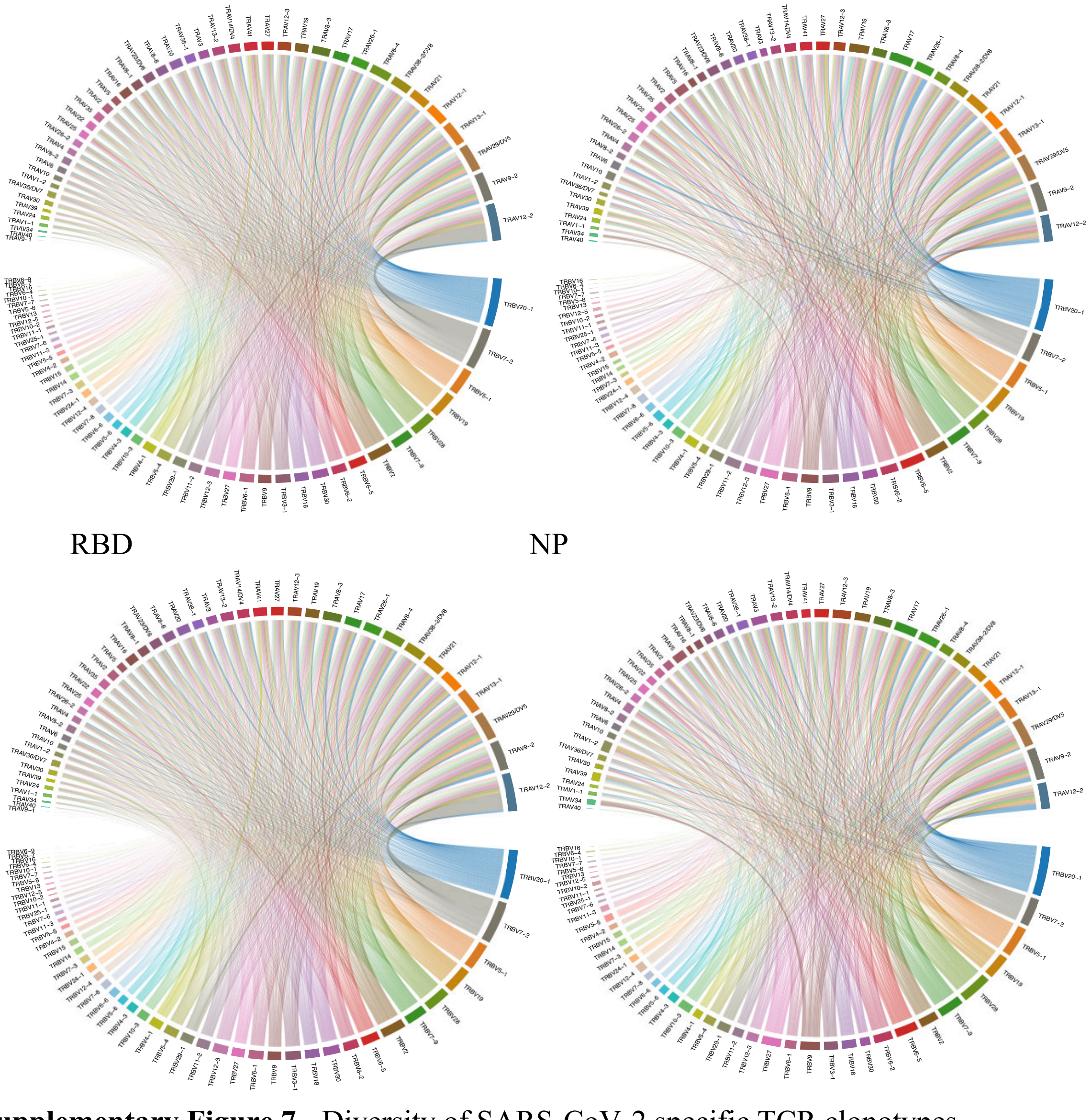

<u>Supplementary Figure 7</u> - Diversity of SARS-CoV-2 specific TCR clonotypes.

(A) Slightly lower Shannon's entropy scores for spike, RBD and NP compared to ex vivo memory.

(B) Circos plot showing high diversity of TCRab usage in all 4 conditions.
